# The relationship between teachers’ critical thinking, experiences of victimization in educational institutions, and psychological well-being

**DOI:** 10.3389/fpsyg.2026.1717632

**Published:** 2026-01-30

**Authors:** Rugile Bitinaite, Aiste Dirzyte, Odeta Merfeldaite, Jolanta Pivoriene, Valdone Indrasiene, Violeta Jegeleviciene, Romas Prakapas, Asta Railiene, Daiva Penkauskienė, Justinas Sadauskas, Marina Gusauskiene

**Affiliations:** Faculty of Human and Social Studies, Mykolas Romeris University, Vilnius, Lithuania

**Keywords:** critical thinking, educational psychology, Lithuania, psychological well-being, teacher victimization

## Abstract

**Introduction:**

Teacher victimization is a growing concern with well-documented implications for educators’ psychological well-being. However, less is known about how such experiences relate not only to emotional outcomes but also to critical thinking. This study aimed to examine the associations between different forms of teacher victimization (e.g., physical, social, verbal, and cyber), psychological well-being (flourishing, positive and negative emotions), and critical thinking.

**Methods:**

Data from 1,044 Lithuanian teachers were collected via an online survey. Victimization, critical thinking, and psychological well-being were measured with validated scales, and structural equation modeling (SEM) was applied to test their interrelationships. Model fit was evaluated using standard indices.

**Results:**

Physical, social, verbal, and cyber victimization were significantly associated with lower psychological well-being. Verbal victimization showed the strongest links, with moderate negative correlations with flourishing (rs = −0.23) and positive emotions (rs = −0.25), and a positive correlation with negative emotions (rs = 0.32, all *p* < 0.001). Social victimization showed similar patterns. Unexpectedly, victimization was weakly but positively related to critical thinking (*β* = 0.07). Higher critical thinking, in turn, predicted lower psychological distress (*β* ≈ −0.27). Mediation analysis indicated a small but significant indirect effect (*β* ≈ −0.02), demonstrating that critical thinking partially mediated the association between victimization and psychological well-being.

**Discussion:**

The findings confirm that teacher victimization adversely affects psychological well-being. At the same time, critical thinking emerged as a meaningful cognitive mechanism: although slightly elevated by victimization, it was associated with lower ill-being, producing a small indirect effect. These findings indicate that critical thinking may play a modest explanatory role in how victimization relates to teachers’ psychological functioning.

## Introduction

Over the past decades, school violence has emerged as a critical public health concern (Astor et al., 2024). While considerable research has examined student victimization, relatively little scholarly attention has been directed toward the victimization of teachers, despite mounting evidence that educators frequently encounter various forms of aggression, including verbal threats, physical attacks, and property damage (Ghadban et al., 2023; McMahon et al., 2024; Reddy et al., 2013). National surveys conducted by the American Psychological Association revealed that up to 80% of school personnel reported verbal or threatening aggression, with a significant proportion also reporting physical violence (McMahon et al., 2024). The consequences of such experiences are profound, often associated with negative outcomes, such as reduced job satisfaction and weakened interpersonal relationships, which may negatively impact teachers’ psychological well-being (Berg and Cornell, 2016; McMahon et al., 2020; Moon and McCluskey, 2018). As violence against educators becomes increasingly recognized as both a public health and educational crisis, it is essential to understand not only its direct psychological effects but also the personal resources that may help mitigate its impact.

One such psychological resource is critical thinking (CT), which has been increasingly recognized as a fundamental competence for navigating the demands of adversarial educational environments (Vincent-Lancrin et al., 2019). Critical thinking enables individuals to analyze complex situations, evaluate evidence, and make reasoned decisions (Facione, 1990; Brookfield, 2017), capacities that are particularly relevant when teachers must respond to challenging or hostile interactions. Research shows that strong critical thinking skills are associated with more adaptive coping, better emotional regulation, and greater psychological resilience when facing stressors or ambiguous social situations (Li et al., 2022; Vosoughi et al., 2025; de Almeida Santos et al., 2018; Mestre et al., 2017; Sk et al., 2024). In educational contexts, CT supports teachers in interpreting difficult classroom dynamics, appraising threats accurately, and selecting constructive rather than reactive responses. These cognitive processes have been linked to improved psychological well-being, as individuals who engage in reflective, analytical thinking tend to experience lower stress, reduced emotional reactivity, and more effective problem-solving when encountering adversity (Agrawal et al., 2023; Daches Cohen et al., 2023; Demirezen and Ötken, 2022; Görücü et al., 2025; Guamanga et al., 2025).

Despite these theoretical connections, little empirical research has examined the role of critical thinking in the context of teacher victimization (TV) (Pivorienė et al., 2024). Given that violence in schools is not only a behavioral problem but also a deeply psychological and relational one, understanding how critical thinking functions as a potential protective factor is an important step toward developing holistic interventions. This study aims to fill this gap by exploring the relationships between teacher victimization, psychological well-being, and critical thinking. In doing so, it seeks to contribute to a broader understanding of how cognitive (interpretation, analysis, evaluation, drawing conclusions, explanation, self-regulation skills) and dispositional (critical openness and reflective skepticism) factors may support teachers in coping with the challenges of their profession and sustaining their well-being in hostile work environments.

### Victimization in educational institutions: prevalence and form

A precise definition of victimization is essential to enhance understanding of its scope and impact within educational contexts. In the scholarly literature, behavior is classified as bullying when it involves repeated acts of aggression, occurs within a context of power asymmetry, and reflects a purposeful intent to harm the target (Cuadrado-Gordillo and Fernández-Ferrer, 2012). In educational research, however, teacher victimization has been operationalized more broadly to encompass both single and repeated incidents of intentional aggression that exploit situational or institutional power asymmetries between teachers and perpetrators (Reddy et al., 2023; McMahon et al., 2024).

Teacher victimization is widely recognized as a multidimensional construct encompassing several distinct forms of aggressive behavior, including verbal, physical, social, property-related, sexual, and cyber manifestations (Yang et al., 2019; Dirzyte et al., 2024; Anderman et al., 2024). Research increasingly recognizes that aggression directed at teachers extends beyond overt physical or verbal attacks to include more subtle and indirect forms of mistreatment. For example, studies have shown that verbal aggression, such as insults, threats, or derogatory comments, is among the most commonly reported experiences and can significantly erode teachers’ sense of professional safety (McMahon et al., 2022; Moon and McCluskey, 2018). Similarly, physical aggression, ranging from pushing or hitting to threats of bodily harm, remains a persistent concern in many school systems globally (Olivier et al., 2021; Yang et al., 2019). In addition to these direct forms, social or relational victimization—including exclusion, rumor-spreading, and deliberate damage to teachers’ reputation—has been identified as a salient yet often under-acknowledged dimension of school-based aggression (Corbett et al., 2022).

Contemporary studies also emphasize the importance of recognizing property-related victimization, such as theft or intentional damage to teachers’ belongings, which may function as symbolic forms of intimidation (McMahon et al., 2023). Moreover, sexual harassment and sexually inappropriate behavior, although less frequently reported, constitute a serious form of victimization with documented psychological consequences for educators (Olivier et al., 2021). The digitalization of school communication has further introduced cyber-based victimization, where teachers experience harassment, threats, or defamatory content mediated through social media or digital platforms (McMahon et al., 2024). Together, these diverse forms illustrate the multidimensional nature of teacher victimization and underscore the need for comprehensive measurement approaches that reflect the full range of behaviors encountered by educators in contemporary school environments. Consistent with recent frameworks (Moon and McCluskey, 2018; Corbett et al., 2022), the present study defines teacher victimization as any intentional act of physical, verbal, social, cyber, sexual, and property-related aggression directed toward teachers, which undermines their professional integrity, emotional safety, or well-being.

Recent research increasingly demonstrates that teacher victimization is a widespread and persistent problem across educational systems worldwide (Dirzyte et al., 2024; Scharpf et al., 2023). Large-scale reviews and national surveys reveal that a substantial proportion of educators—often more than half in many regions—report experiencing some form of victimization during the school year, underscoring its status as a critical occupational concern rather than an exceptional occurrence (Longobardi et al., 2019; McMahon et al., 2024). Contemporary studies across diverse international settings similarly point to high rates of psychological, verbal, and physical victimization among teachers, with prevalence estimates ranging from moderate to severe in both primary and secondary schools (Dirzyte et al., 2024; Andersen et al., 2025). Large-scale studies in Europe and Asia echo these trends: in a Lithuanian sample, 65.8% of teachers reported victimization by students, 33.9% verbal victimization by parents, and 38.5% bullying by school staff (Dirzyte et al., 2024), while a Danish follow-up study found that 62% of teachers had experienced harassment, 41% threats, and 39% physical violence from pupils in the previous year (Andersen et al., 2025). In China, Yang et al. (2019) reported that 25.1% of secondary teachers experienced at least one form of victimization (physical, verbal, social, cyber, sexual harassment, or property offenses) from students in the past school year, with social victimization most prevalent and male and homeroom teachers at greater risk. Complementing these primary studies, a recent systematic review concluded that client-initiated violence against educational workers—including teachers and other school staff—is a global problem characterized by substantial rates of physical, psychological, and verbal assaults (Calderon-Orellana et al., 2025). Together, these findings underscore the alarming prevalence and multifaceted nature of teacher victimization worldwide, and highlight its implications for educators’ well-being and career decisions.

Given the widespread and persistent nature of teacher victimization, its consequences for educators’ professional and psychological functioning have become an urgent focus of recent scholarship. Teachers’ exposure to multiple forms of victimization—including theft or property damage, physical assault, verbal abuse, sexual harassment, and noncontact aggression—has been shown to undermine key aspects of their work experiences, such as perceived job performance, trust in students, feelings of safety, and intentions to remain in their positions (McMahon et al., 2022; Moon and McCluskey, 2018; Olivier et al., 2021). National-level evidence further demonstrates that verbal and physical violence substantially elevates teacher anxiety and stress, which in turn strongly predict teachers’ intentions to transfer schools or leave the profession altogether; anxiety and stress serve as critical mediators linking violent encounters to turnover-related decisions (McMahon et al., 2023). Such findings are consistent with broader research showing that teacher-directed aggression negatively affects staff well-being, erodes teachers’ sense of belonging within their school communities, reduces job satisfaction, and contributes to heightened attrition rates (McMahon et al., 2024; Corbett et al., 2022; Moon et al., 2021).

In addition to these detrimental outcomes, research shows that both contextual and individual factors shape teachers’ vulnerability to victimization. School climate, organizational conditions, and community characteristics are significant predictors of aggression toward teachers (Reddy et al., 2018, 2023). At the same time, teacher characteristics also play an important role: demographic variables such as gender, experience level, and subject area have been linked to differential rates of violence, with male and less-experienced teachers often reporting higher victimization (Moon and McCluskey, 2018; Berlanda et al., 2019; Anderman et al., 2024). Cognitive and relational qualities—including attributional styles, relationship-building approaches, and classroom management skills—further contribute to risk, as teachers who exhibit higher self-blame or weaker management skills tend to encounter more frequent or severe incidents (Martinez et al., 2016; Espelage et al., 2013; American Psychological Association, 2013; Wiley et al., 2012). Understanding how these qualities interact with contextual factors is therefore essential for developing targeted training, prevention efforts, and supports to reduce victimization and promote teacher resilience (Reddy et al., 2018; Berlanda et al., 2019).

### The concept of critical thinking and its complexity

Although critical thinking has been extensively examined, it remains a complex and multifaceted construct that is conceptualized differently across disciplines such as psychology, philosophy, and education (Ellerton et al., 2024). These disciplinary perspectives produce varying interpretations of the concept; however, despite their differences, most scholars associate critical thinking with sound reasoning (Johnson and Hamby, 2015) and reflective, innovative, and independent thought (Willingham, 2020), which enables individuals to make justified and context-sensitive decisions (Rodzalan and Saat, 2015; Ennis, 2011; Facione, 1990, 2011; Halpern, 1998).

Across theoretical models developed by different scholars, critical thinking is generally understood as comprising two essential and closely interrelated components: Critical Thinking Skills and Critical Thinking Dispositions (Payan-Carreira et al., 2022). Although the skills identified by various authors (Paul and Elder, 2020; Elder et al., 2007, Beyer, 1987; Ennis, 1987; Halpern, 1998; Barnett, 1997; Facione, 1990) often overlap—typically including interpretation, analysis, and evaluation—the associated dispositions are framed in more diverse ways, ranging from moral and intellectual virtues to self-reflection and existential critique. Paul and Elder (2020) and Elder et al. (2007) framework presents critical thinking as stemming from the integration of cognitive abilities and value-laden dispositions that foster intellectual virtues and continual improvement of thought processes, with particular emphasis on learning and reflection as foundations for enhancing reasoning quality. Other frameworks of critical thinking foreground different aspects of dispositions: highlights systematic and disciplined information processing, coupled with caution and critical scrutiny (Beyer, 1987); stresses that critical thinking requires not only the ability but also the disposition—the willingness—to be fair-minded, open, and precise in one’s judgments (Ennis, 1987); expands the list of critical thinking skills, underscoring problem solving and argument analysis, while placing special emphasis on motivation, self-regulation, and communication (Halpern, 1998); adds an existential dimension by encouraging reflection on the nature of knowledge and social practices and situating dispositions within a broader context of life-wide self-reflection (Barnett, 1997). Facione (1990) conceptualizes critical thinking as a constellation of interrelated skills—interpretation, analysis, evaluation, inference, explanation, and self-regulation—and dispositions, emphasizing that dispositions are essential for the effective application of skills.

Facione’s (1990) model is widely regarded as one of the most coherent and empirically grounded frameworks of critical thinking, integrating both cognitive and dispositional components. Compared with other well-established perspectives, the Facione model is more paradigmatically neutral, more readily operationalized, and provides clearer differentiation between cognitive competencies and dispositions, making it particularly suitable for empirical research. Accordingly, the present study adopts Facione’s (1990) conceptualization of critical thinking - which also informed the adaptation of the study’s instruments - and focuses on six skills (interpretation, analysis, evaluation, inference, explanation, and self-regulation) and two dispositions: critical openness and reflective skepticism, both of which encompass broader dispositions such as open-mindedness and cognitive flexibility.

Given that critical thinking is an integrated construct that unites cognitive skills and dispositions within a dynamic, context-dependent process of reflection and learning, it enables individuals to reflect systematically, self-regulate, and make reasoned decisions (Jaramillo Gómez et al., 2025; Indrašienė et al., 2021). This conceptualization of critical thinking as reflective and analytical thought is particularly relevant in the context of traumatic experiences such as victimization. Research has shown that cognitive appraisals are key mechanisms linking traumatic events with mental health outcomes (Beck and Haigh, 2014; Schäfer et al., 2017). Critical thinking skills - such as analysis, evaluation, inference, and self-regulation—may help individuals critically examine initial beliefs, correct maladaptive appraisals, and develop more adaptive interpretations of stressful events (Facione, 1990; Halpern, 2014). Studies indicate that reflective, structured thinking and meaningful interpretation of events contribute to reduced psychological distress and foster post-traumatic growth (Park, 2010; Pals and McAdams, 2004).

Accordingly, critical thinking might be construed as a potential mediating process that could enable teachers to navigate experiences of aggression, threats, or psychological pressure in their professional practice. It helps explain how teachers analyze and interpret complex classroom social dynamics, evaluate contextual factors such as discrimination, bullying, or power relations, and ultimately respond more constructively to challenges while maintaining professional composure and psychological well-being (Ten Dam and Volman, 2004). Through reflective and non-judgmental appraisal, critical thinking mediates teachers’ tendencies to interpret students’ misbehavior as personal attacks (Jennings et al., 2017). Teachers who apply critical thinking also model critical stances—demonstrating openness, reflection, problem recognition, and intolerance of dishonesty or harmful actions (Peist et al., 2020). In this sense, Facione’s (1990) skills—particularly analysis, evaluation, and self-regulation—act as the processes through which teachers interpret incidents more objectively, identify faulty assumptions, and adopt adaptive response strategies, while critical thinking dispositions mediate the maintenance of an impartial, flexible, and deliberate perspective (Ellerton, 2022). Taken together, these theoretical and empirical insights suggest that critical thinking functions as a process shaped by experience and deployed in response to professional adversity. This raises the possibility that exposure to victimization may influence teachers’ reflective and evaluative processes, which subsequently shape their psychological adjustment. In the present study, critical thinking is therefore conceptualized and tested as a mediator: a mechanism through which victimization experiences may affect teachers’ psychological well-being, by altering how they interpret, analyze, and respond to challenging interpersonal and organizational dynamics.

### Teachers’ psychological well-being

Teachers’ psychological well-being is a central construct in understanding how educators interpret, endure, and respond to adverse experiences in their professional environment. Rather than functioning as a broad, generic concept, psychological well-being provides a meaningful lens through which to examine teachers’ vulnerability to stressors such as victimization and their capacity to draw on cognitive resources—such as critical thinking—when navigating these challenges. Well-being is broadly conceptualized as an interplay of personal, social, and contextual factors that shape individuals’ sense of meaning, identity, and functioning (McCallum and Price, 2015). Within psychological science, two complementary perspectives help clarify its relevance. The hedonic approach emphasizes subjective experiences of affect and life satisfaction (Ryan and Deci, 2001), whereas the eudaimonic perspective highlights psychological functioning, personal growth, and fulfillment of basic psychological needs. Both perspectives are essential for understanding how teachers’ well-being is disrupted by occupational stressors and supported through internal strengths.

Importantly for the present study, psychological well-being is not merely the absence of distress but reflects the presence of adaptive emotional and cognitive functioning—including positive affect, life satisfaction, and resilience (Keyes, 2013). Higher well-being supports teachers’ ability to regulate emotions, cope constructively with stress, maintain motivation, and sustain professional engagement despite adversity (Obrenovic et al., 2020; Fletcher and Sarkar, 2016). These processes are highly relevant to situations of victimization, where teachers’ psychological resources may determine whether they respond reactively or engage in more reflective, problem-focused strategies. Evidence-based interventions such as mindfulness, cognitive-behavioral strategies, and social–emotional learning have been shown to enhance psychological well-being by reducing emotional exhaustion and strengthening coping capacities (Keng et al., 2011; Renshaw et al., 2015). This underscores the modifiable nature of well-being and its potential as a protective factor in stressful educational environments.

Teacher well-being also emerges from workplace conditions and the broader organizational environment. In educational settings, it has been defined as an individual sense of professional purpose, satisfaction, and relational connectedness (Acton and Glasgow, 2015). Frameworks proposed by Van Horn et al. (2004) and McCallum and Price (2015) highlight the multidimensional nature of professional well-being, encompassing affective, social, cognitive, professional, and psychosomatic domains. These dimensions reflect the complex and interdependent factors that sustain or erode teachers’ psychological functioning. Empirical studies show that job demands, job control, and social support significantly predict well-being (Ibrahim et al., 2021), while chronic exposure to stress-inducing conditions can undermine teachers’ motivation, confidence, and commitment to the profession (Viac and Fraser, 2020; Collie et al., 2012; Desrumaux et al., 2015). Teachers experiencing sustained stress or emotionally difficult interactions—including victimization—are more likely to report heightened negative affect, reduced flourishing, and increased symptoms of ill-being such as anxiety or depressive states. Additional risk factors include age, long teaching tenure, heavy workloads, and elevated psychological demands (Desouky and Allam, 2017; Hadi et al., 2009). Organizational features such as school leadership, professional culture, and access to meaningful development opportunities further shape teachers’ psychological well-being by influencing their sense of belonging, autonomy, and support (Day et al., 2006).

Taken together, these findings underscore that teachers’ psychological well-being is shaped by the dynamic interplay of personal resources and workplace stressors, making it a critical factor in understanding how educators interpret and respond to adverse experiences such as victimization. As teachers’ well-being is related to their coping behaviors, motivation, and long-term occupational functioning—it reinforces the importance of examining psychological well-being within contexts of chronic stress and demanding professional environments (Song, 2022; Li et al., 2022; Dreer and Gouasé, 2023).

### Present study

Teacher victimization, encompassing verbal, physical, and psychological aggression within educational environments, has been increasingly recognized as a significant occupational hazard with detrimental effects on educators’ psychological well-being and job satisfaction (McMahon et al., 2023; Reddy et al., 2023). While prior research has extensively documented the prevalence and consequences of victimization, considerably less attention has been devoted to understanding how victimization may influence teachers’ internal cognitive functioning and, consequently, their psychological well-being.

While much research has focused on the emotional and psychological consequences of victimization, less attention has been directed toward the cognitive processes that may influence teachers’ responses to such experiences. These gaps point to the need to consider the cognitive resources teachers bring to challenging situations. Critical thinking—defined as the ability to analyze, evaluate, and synthesize information in a reflective and objective manner—is widely regarded as a core cognitive competency for educators, supporting effective decision-making and problem-solving in complex school environments (Payan-Carreira et al., 2024; Sosu, 2013). Although critical thinking is often assumed to contribute to more adaptive appraisal and coping processes, existing empirical work has not examined whether experiences of victimization might undermine teachers’ critical thinking capacities and thereby negatively affect their well-being. Thus, rather than conceptualizing critical thinking as a fixed protective factor that buffers victimization, the present study approaches it as a potential mechanism through which victimization may influence psychological functioning. This aligns with theoretical perspectives suggesting that exposure to persistent stressors can impede higher-order cognitive processes, with implications for emotional and psychological adjustment.

A recent mapping review confirmed that, despite extensive research on each construct—critical thinking, psychological well-being, and teacher victimization—their interrelationships remain largely unexplored. Prior studies have predominantly focused on the direct association between victimization and diminished well-being, while connections between victimization and critical thinking, and between critical thinking and well-being, have received minimal empirical attention (Pivorienė et al., 2024). Notably, no studies to date have tested whether critical thinking serves as an explanatory mechanism linking victimization to negative psychological outcomes among teachers.

To address this gap, the present study examines the associations among teacher victimization, critical thinking, and psychological well-being. Specifically, it tests whether critical thinking mediates the relationship between experiences of victimization and negative psychological well-being (ill-being). The study is guided by the following hypotheses:

*H1*: Teacher victimization is positively associated with negative psychological well-being; higher levels of victimization will predict greater psychological distress.

*H2*: Teacher victimization is negatively associated with critical thinking; as exposure to victimization increases, critical thinking dispositions and skills will decrease.

*H3*: Critical thinking is negatively associated with psychological ill-being; teachers with stronger critical thinking skills will report lower psychological distress.

*H4*: Critical thinking mediates the relationship between victimization and negative psychological well-being, such that victimization exerts an indirect positive effect on distress through its negative impact on critical thinking.

Structural equation modeling (SEM) was employed to test both the measurement and structural components of the model. Based on theoretical and empirical considerations, it was expected that (1) the seven observed forms of victimization (parental, property, sexual, cyber, verbal, social, physical) would load on a higher-order Teacher Victimization factor; (2) all eight indicators of critical thinking (two dispositions and six skills) would load on a single Critical Thinking factor; (3) a more parsimonious critical thinking factor would emerge after removing the two dispositional indicators; and (4) negative emotions would load positively, and positive emotions and flourishing would load negatively (inverse valence), on a single Negative Psychological Well-Being factor.

By integrating these constructs within a single analytic framework, the present study contributes to a more comprehensive understanding of how cognitive processes may be affected by victimization and how such effects, in turn, may shape teachers’ psychological well-being. This approach offers a theoretical and empirical foundation for developing interventions aimed not only at reducing victimization but also at strengthening teachers’ cognitive capacities to support their long-term professional functioning.

## Method

### The sample

This study received ethical approval from the Scientific Committee of the Lifelong Learning Laboratory at Mykolas Romeris University (Protocol No. 10-79 2.25 E-403) on February 25, 2025, ensuring compliance with ethical research standards. The research forms part of a larger project examining victimization experiences and well-being among teachers in Lithuania.

The data were collected using a computer-assisted online survey, which required approximately 30 min to complete. Participation was entirely voluntary and anonymous, and respondents did not receive any incentives for their involvement. Participation in the study was voluntary and anonymous, and all participants were free to withdraw from the study at any time. The online questionnaire was implemented using a mandatory-response format for the core study items, meaning that respondents could not proceed to the next question without selecting an answer option. This technical setting was introduced to minimize partial responses and to avoid the need for data imputation. It did not restrict participants’ autonomy, as they could choose not to participate in the study or discontinue participation at any point by closing the survey window, without any negative consequences. This approach is consistent with the protocol approved by the Ethics Committee and with general principles of research ethics. The principles of fairness and absence of bias were observed during the conduct of the study and the processing of data (British Educational Research Association, 2024). In addition, the ethical principles of the study were based on the provisions of the European Code of Conduct for Researchers, which emphasizes responsibility for research quality and ethical behavior (ALLEA, 2023).

A total of 1,044 teachers working in educational institutions across various Lithuanian cities participated in the study. The majority of participants were women (90.1%, *n* = 941), while men comprised 9.3% (*n* = 97). One participant identified as “Other” (0.1%), and gender information was missing for five individuals (0.5%). Participants’ ages ranged from 20 to 72 years, with a mean age of 52.2 years (SD = 9.74). This gender distribution is broadly consistent with the Lithuanian population of general education teachers and school leaders, among whom women constitute 88.3% (24,016 out of 27,187) and men only 11.7% (3,171).

In terms of teaching roles, 25.1% (*n* = 262) were primary education teachers, while 74.9% (*n* = 782) were subject teachers. Furthermore, 44.0% (*n* = 459) had more than 30 years of teaching experience. Regarding educational attainment, most participants held either a bachelor’s degree (53.1%) or a master’s degree (43.6%). In terms of marital status, the majority reported living with a spouse or partner, either with children (34.7%) or without (41.7%).

### Instruments

To measure teachers’ experienced victimization, the Multidimensional Teacher Victimization Scale (MTVS) (Yang et al., 2019), with supplementary items added by the study authors, was employed. The MTVS comprises multiple items designed to capture a wide range of victimization types including physical, verbal, social, cyber, sexual, and property-related aggression. Each item is rated on a 5-point Likert scale reflecting frequency, which allows for detailed quantification of victimization experiences. This instrument has demonstrated solid psychometric properties, such as reliability and validity, across previous studies involving educator populations. Additional questions were also included to capture victimization perpetrated by students’ parents.

To assess critical thinking dispositions, the study employed two validated scales. The Critical Thinking Dispositions Scale (Sosu, 2013) was used to evaluate teachers’ tendencies toward reflective, analytical, and open-minded thinking. This scale utilizes a Likert-type response format to capture the strength of critical thinking traits essential for effective professional judgment. It was developed in response to limitations identified in the widely used California Critical Thinking Disposition Inventory (CCTDI; Facione and Facione, 1992), as cross-validation studies revealed inconsistencies in item loadings, excessive inter-item correlations, conceptual overlap, and an unstable factor structure. Consequently, Sosu’s scale isolates two refined dispositions: critical openness and reflective skepticism, which together encompass 11 items.

Complementing this, the Critical Thinking Self-Assessment Scale – Short Form (Payan-Carreira et al., 2024) provides a concise measurement of self-perceived critical thinking skills. This scale was originally developed by Nair and validated by Payan-Carreira et al. (2024), aligning with six skills of Facione’s critical thinking framework: interpretation, analysis, evaluation, inference, explanation, and self-regulation. It focuses particularly on metacognitive abilities and reasoning confidence, capturing problem-solving, reasoning, and criticality skills that are integral to effective decision-making.

Psychological well-being was measured using two well-established instruments. The Flourishing Scale (Diener et al., 2011) captures overall positive functioning by assessing key domains such as meaning, engagement, and optimism. It employs an 8-item Likert scale, with higher scores indicating greater well-being and life satisfaction. Additionally, the Positive and Negative Affect Schedule (PANAS) (Watson et al., 1988) was administered to measure both positive and negative emotional states. Composed of two 10-item subscales, the PANAS uses Likert-type responses to assess the frequency of specific feelings over a given time frame, thereby offering insight into teachers’ affective experiences.

### Statistical analysis

Prior to conducting the statistical analyzes, the dataset was screened for data quality and completeness. As the online questionnaire was configured so that all substantive items required a response before proceeding, all submitted questionnaires contained complete answers on the study variables. Consequently, there were no missing values on the variables included in the analyzes, no imputation procedures were required, and all tests were performed on the full analytic sample.

All statistical analyzes were performed using SPSS (Version 29.0) and AMOS software (Arbuckle, 2022). Descriptive statistics, including means, standard deviations, skewness, and kurtosis, were Hcalculated to assess the distributional properties of the study variables.

To examine bivariate relationships between teacher victimization, critical thinking, and psychological well-being, Spearman’s rank-order correlation coefficients were computed. This nonparametric approach was selected due to non-normality in the distribution of several variables, as indicated by elevated skewness and kurtosis values (Tabachnick and Fidell, 2014).

Structural equation modeling (SEM) was employed to test the hypothesized associations among teacher victimization, critical thinking, and psychological well-being. Two competing models were estimated: Model 1 included all eight indicators of critical thinking (both dispositional and skill-based), while Model 2 included only six skill-based indicators to improve the model. Model fit was evaluated using multiple fit indices: chi-square (χ^2^), Comparative Fit Index (CFI), Tucker–Lewis Index (TLI), Root Mean Square Error of Approximation (RMSEA) with 90% confidence intervals, Standardized Root Mean Square Residual (SRMR), and Akaike Information Criterion (AIC), in line with recommended reporting standards (Kline, 2016).

Unstandardized regression coefficients (B), standard errors (SE), critical ratios (CR), standardized path coefficients (*β*), and associated *p*-values were reported for all structural paths. The proportion of explained variance (R^2^) was also calculated for latent constructs. Statistical significance was determined using a threshold of *p* < 0.05. Indirect effects were examined to test for mediation in accordance with contemporary SEM practices (Kline, 2016; Rosseel, 2012).

## Results

### Descriptive statistics and correlations

To explore the associations among key study variables, preliminary analyzes were conducted to assess distributional assumptions and select appropriate statistical methods. Given significant violations of normality, evidenced by extreme skewness and kurtosis in several variables, a non-parametric method was deemed appropriate. Descriptive statistics are presented in Table 1. Accordingly, a series of Spearman’s rank-order correlations were conducted to examine the relationships between different types of teacher victimization, psychological well-being, and critical thinking; the results of these correlations are presented in Table 2.

**Table 1:** Descriptive statistics and normality indicators for teacher victimization, psychological well-being, and critical thinking variables.

Parameters	Mean	Std. Deviation	Skewness	Kurtosis
Physical TV	1.0805	0.29394	8.008	84.027
Social TV	1.3776	0.64194	2.667	8.873
Verbal TV	1.3242	0.55032	3.066	12.588
Cyber TV	1.0656	0.30965	9.344	104.534
Sexual TV	1.1454	0.35542	5.482	44.867
Property TV	1.1027	0.31489	6.139	51.196
Parental TV	1.1410	0.37163	4.783	30.326
Flourishing	6.1934	0.81145	−2.573	11.571
Positive motions	3.8706	0.66880	−0.687	0.845
Negative motions	2.1375	0.72019	0.676	0.357
Critical openness	4.0517	0.54286	−0.461	1.806
Reflective scepticism	4.1655	0.59064	−0.642	1.520
Interpretation	5.2533	0.95636	−0.414	0.310
Analysis	5.1654	1.10414	−0.499	0.531
Evaluation	5.2295	1.16027	−0.701	0.766
Drawing conclusions	5.1899	1.14766	−0.656	0.738
Explanation	5.2581	1.08743	−0.681	1.010
Self-regulation	5.2963	1.11208	−0.657	0.649

**Table 2:** Spearman’s correlations between teacher victimization, psychological well-being, and critical thinking variables.

Correlations	Spearman’s rho	*p*	Effect size (Fisher’s z)	Covariance
Physical TV – Flourishing	−0.094**	0.002	−0.094	−0.022
Physical TV – Positive emotions	−0.111***	< 0.001	−0.112	−0.025
Physical TV – Negative emotions	0.164***	< 0.001	0.166	0.044
Physical TV – Critical openness	0.053	0.086	0.053	0.005
Physical TV – Reflective scepticism	0.057	0.065	0.057	<0.001
Physical TV – Interpretation	0.038	0.215	0.038	0.006
Physical TV – Analysis	0.038	0.216	0.038	0.014
Physical TV – Evaluation	0.055	0.074	0.055	0.017
Physical TV – Drawing conclusions	0.044	0.159	0.044	0.022
Physical TV – Explanation	0.040	0.194	0.040	0.022
Physical TV – Self – regulation	0.040	0.195	0.040	0.021
Social TV – Flourishing	−0.193***	< 0.001	−0.196	−0.062
Social TV – Positive emotions	−0.223***	< 0.001	−0.227	−0.095
Social TV – Negative emotions	0.297***	< 0.001	0.306	0.141
Social TV – Critical openness	−0.061*	0.048	−0.061	0.005
Social TV – Reflective scepticism	−0.044	0.153	−0.044	0.010
Social TV – Interpretation	−0.001	0.962	−0.001	0.036
Social TV – Analysis	−0.005	0.884	−0.005	0.046
Social TV – Evaluation	0.004	0.893	0.004	0.053
Social TV – Drawing conclusions	−0.009	0.762	−0.009	0.053
Social TV – Explanation	−0.016	0.613	−0.016	0.051
Social TV – Self – regulation	−0.022	0.482	−0.022	0.041
Verbal TV – Flourishing	−0.227***	< 0.001	−0.231	−0.068
Verbal TV – Positive emotions	−0.253***	< 0.001	−0.259	−0.084
Verbal TV – Negative emotions	0.323***	< 0.001	0.335	0.129
Verbal TV – Critical openness	−0.064*	0.037	−0.065	<0.001
Verbal TV – Reflective scepticism	−0.062*	0.045	−0.062	0.003
Verbal TV – Interpretation	−0.012	0.705	−0.012	0.028
Verbal TV – Analysis	−0.022	0.482	−0.022	0.035
Verbal TV – Evaluation	0.017	0.593	0.017	0.047
Verbal TV – Drawing conclusions	0.002	0.954	0.002	0.044
Verbal TV – Explanation	0.002	0.947	0.002	0.041
Verbal TV – Self – regulation	−0.022	0.476	−0.022	0.030
Cyber TV – Flourishing	−0.118***	< 0.001	−0.119	−0.023
Cyber TV – Positive emotions	−0.165***	< 0.001	−0.166	−0.018
Cyber TV – Negative emotions	0.201***	< 0.001	0.204	0.042
Cyber TV – Critical openness	−0.044	0.154	−0.044	<0.001
Cyber TV – Reflective scepticism	−0.023	0.458	−0.023	−0.003
Cyber TV – Interpretation	−0.013	0.665	−0.013	−0.001
Cyber TV – Analysis	−0.005	0.880	−0.005	0.008
Cyber TV – Evaluation	0.005	0.865	0.005	0.009
Cyber TV – Drawing conclusions	0.020	0.528	0.020	0.017
Cyber TV – Explanation	0.034	0.270	0.034	0.017
Cyber TV – Self – regulation	0.009	0.766	0.009	0.011
Sexual TV – Flourishing	−0.127***	< 0.001	−0.128	−0.031
Sexual TV – Positive emotions	−0.176***	< 0.001	−0.178	−0.042
Sexual TV – Negative emotions	0.214***	< 0.001	0.218	0.063
Sexual TV – Critical openness	−0.010	0.752	−0.010	0.001
Sexual TV – Reflective scepticism	0.011	0.711	0.011	0.004
Sexual TV – Interpretation	<0.001	0.978	<0.001	0.007
Sexual TV – Analysis	−0.015	0.623	−0.015	0.014
Sexual TV – Evaluation	<0.001	0.991	<0.001	0.015
Sexual TV – Drawing conclusions	−0.015	0.625	−0.015	0.018
Sexual TV – Explanation	−0.007	0.829	−0.007	0.013
Sexual TV – Self – regulation	−0.011	0.728	−0.011	0.018
Property TV – Flourishing	−0.187***	< 0.001	−0.189	−0.032
Property TV – Positive emotions	−0.185***	< 0.001	−0.188	−0.032
Property TV – Negative emotions	0.173***	< 0.001	0.175	0.049
Property TV – Critical openness	−0.022	0.474	−0.022	0.002
Property TV – Reflective scepticism	−0.026	0.405	−0.026	<0.001
Property TV – Interpretation	−0.022	0.478	−0.022	<0.001
Property TV – Analysis	−0.031	0.311	−0.031	0.007
Property TV – Evaluation	−0.008	0.807	−0.008	0.014
Property TV – Drawing conclusions	−0.026	0.398	−0.026	0.010
Property TV – Explanation	−0.017	0.585	−0.017	0.013
Property TV – Self – regulation	−0.021	0.503	−0.021	0.013
Parental TV – Flourishing	−0.102***	< 0.001	−0.102	−0.026
Parental TV – Positive emotions	−0.128***	< 0.001	−0.129	−0.039
Parental TV – Negative emotions	0.189***	< 0.001	0.191	0.064
Parental TV – Critical openness	−0.025	0.424	−0.025	<0.001
Parental TV – Reflective scepticism	−0.030	0.337	−0.030	<0.001
Parental TV – Interpretation	0.013	0.666	0.013	0.009
Parental TV – Analysis	0.025	0.418	0.025	0.020
Parental TV – Evaluation	0.047	0.129	0.047	0.030
Parental TV – Drawing conclusions	0.027	0.377	0.027	0.028
Parental TV – Explanation	0.025	0.420	0.025	0.029
Parental TV – Self – regulation	0.018	0.569	0.018	0.028
Flourishing – Self – regulation	0.337***	< 0.001	0.350	0.216
Positive emotions – Negative emotions	−0.635***	< 0.001	−0.749	−0.312
Positive emotions – Critical openness	0.340***	< 0.001	0.355	0.107
Positive emotions – Reflective scepticism	0.305***	< 0.001	0.315	0.106
Positive emotions – Interpretation	0.280***	< 0.001	0.287	0.158
Positive emotions – Analysis	0.272***	< 0.001	0.279	0.170
Positive emotions – Evaluation	0.243***	< 0.001	0.248	0.163
Positive emotions – Drawing conclusions	0.246***	< 0.001	0.251	0.161
Positive emotions – Explanation	0.275***	< 0.001	0.282	0.173
Positive emotions – Self – regulation	0.296***	< 0.001	0.305	0.187
Negative emotions – Critical openness	−0.196***	< 0.001	−0.198	−0.058
Negative emotions – Reflective scepticism	−0.182***	< 0.001	−0.184	−0.062
Negative emotions – Interpretation	−0.110***	< 0.001	−0.111	−0.043
Negative emotions – Analysis	−0.126***	< 0.001	−0.127	−0.062
Negative emotions – Evaluation	−0.102***	< 0.001	−0.102	−0.050
Negative emotions – Drawing conclusions	−0.124***	< 0.001	−0.124	−0.069
Negative emotions – Explanation	−0.139***	< 0.001	−0.140	−0.073
Negative emotions – Self – regulation	−0.158***	< 0.001	−0.160	−0.092

*p* < 0.05*, *p* < 0.01**, *p* < 0.001***.

Before interpreting the correlation results, it is important to note that both the flourishing and positive emotions variables were reverse scored, such that higher scores indicate lower well-being or less positive emotional experience. As a result, negative correlations with these variables reflect poorer outcomes, while positive correlations indicate better outcomes.

Results revealed that physical victimization was significantly associated with lower emotional well-being, showing small negative correlations with flourishing (rs = −0.09, *p* = 0.002) and positive emotions (rs = −0.11, *p* < 0.001), and a positive correlation with negative emotions (rs = 0.16, *p* < 0.001). Social victimization demonstrated stronger negative correlations with flourishing (rs = −0.19, *p* < 0.001) and positive emotions (rs = −0.22, *p* < 0.001), and a stronger positive correlation with negative emotions (rs = 0.30, *p* < 0.001). Verbal and cyber victimization showed similar patterns with verbal victimization exhibiting the strongest associations (flourishing rs = −0.23, positive emotions rs = −0.25, negative emotions rs = 0.32, all *p* < 0.001).

Flourishing was positively correlated with critical openness (rs = 0.38, *p* < 0.001), reflective scepticism (rs = 0.34, *p* < 0.001), and other critical thinking skills (rs ≈ 0.30–0.34, all *p* < 0.001). Positive emotions demonstrated comparable positive correlations, while negative emotions were negatively correlated with these cognitive variables (rs ranging from −0.10 to −0.20, all *p* < 0.001).

Together, these results indicate that greater exposure to teacher victimization, particularly in the form of verbal, social, and cyber victimization, is consistently associated with lower levels of emotional well-being and increased experience of negative emotions. Furthermore, individuals reporting higher levels of flourishing and positive affect also tended to demonstrate stronger critical thinking dispositions and emotional self-regulation, while those experiencing more negative emotions showed reduced engagement in these cognitive-affective skills.

### Measurement and structural models

To test H1–H4, two structural equation models were estimated in SPSS v29, AMOS. In both, teacher victimization was a second-order latent construct with seven first-order indicators (parental, property, sexual, cyber, verbal, social and physical victimization). Psychological well-being (PWB) was conceptualized as negative well-being formed by three variables: negative emotions (NE), positive emotions (PE) and flourishing (FL). Larger scores on the latent negative PWB factor therefore correspond to greater psychological ill-being. Critical thinking (CT) was specified as a single first-order factor. Model 1 (“full CT”) retained the full eight indicators (critical openness, reflective skepticism, interpretation, analysis, evaluation, drawing conclusions, explanation, self-regulation), Model 2 (“skills-only CT”) dropped the two dispositional indicators (critical openness, reflective skepticism) on the basis of lower loadings and large modification indices.

**Figure 1:**
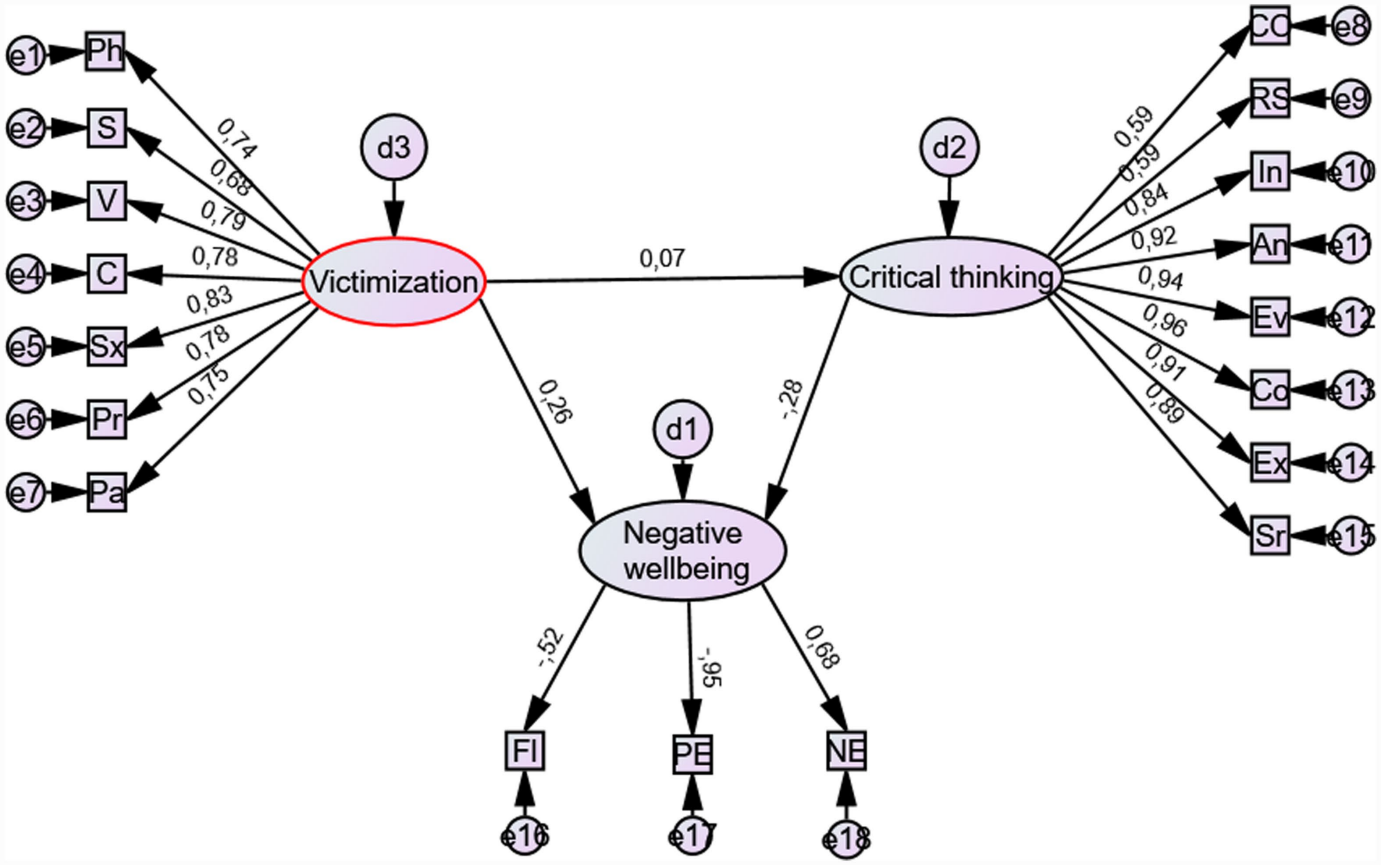
SEM model 1 of associations between teacher victimization, critical thinking, and psychological well-being. FL = flourishing (reverse scored), PE = positive emotions (reverse scored), NE = negative emotions, CO = critical openness, RS = reflective skepticism, In = interpretation, An = analysis, Ev = evaluation, Co = drawing conclusions, Ex = explanation, Sr. = self-regulation, Pa = parental victimization, Pr = property victimization, Sx = sexual victimization, C = cyber victimization, V = verbal victimization, S = social victimization, Ph = physical victimization.

Figure 1 presents Model 1, which includes eight indicators of critical thinking.

**Figure 2:**
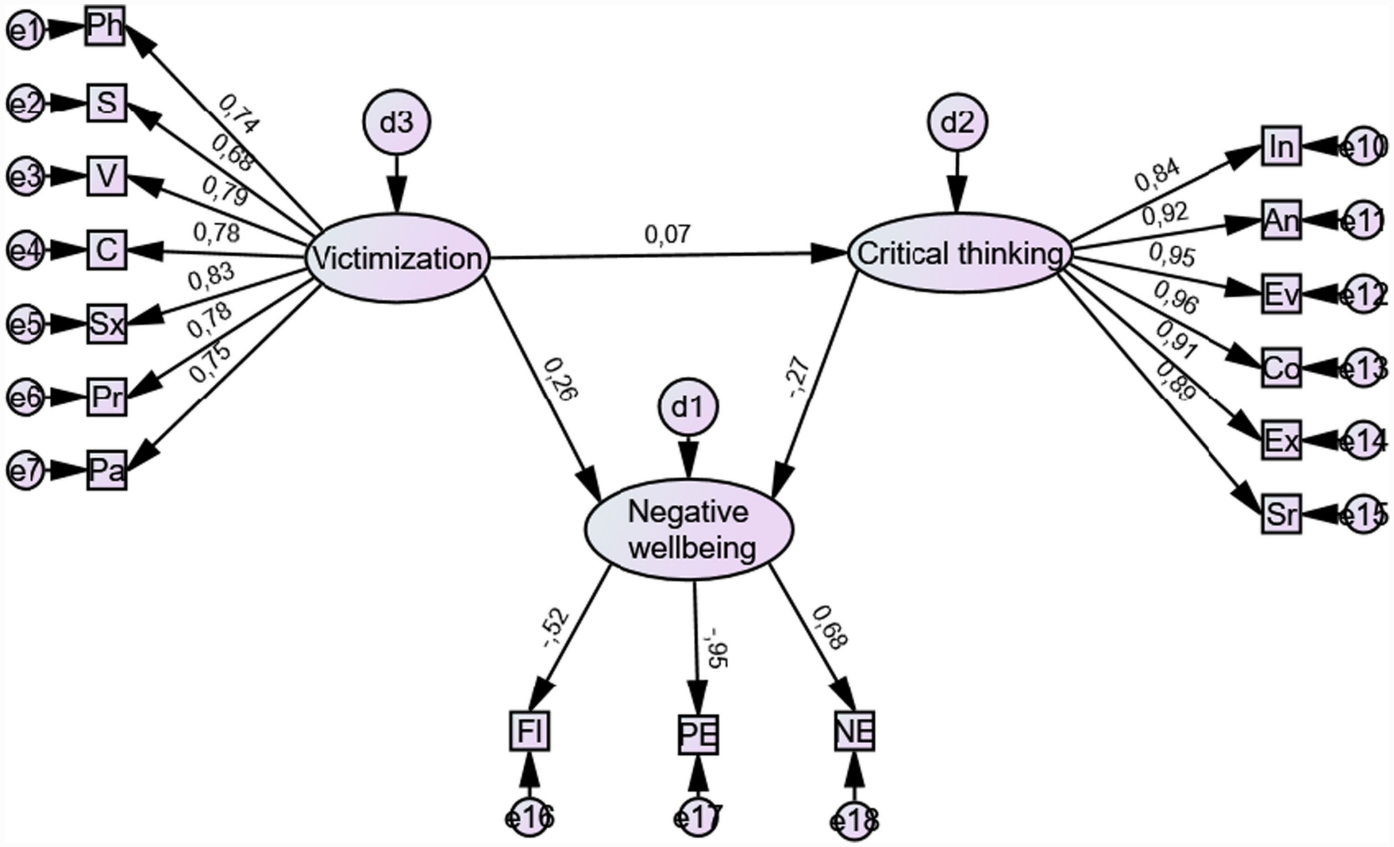
SEM model 2 of associations between teacher victimization, critical thinking, and psychological well-being. FL = flourishing (reverse scored), PE = positive emotions (reverse scored), NE = negative emotions, In = interpretation, An = analysis, Ev = evaluation, Co = drawing conclusions, Ex = explanation, Sr. = self-regulation, Pa = parental victimization, Pr = property victimization, Sx = sexual victimization, C = cyber victimization, V = verbal victimization, S = social victimization, Ph = physical victimization.

Figure 2 presents Model 2, which includes six indicators of critical thinking.

Table 3 summarizes global fit statistics. Model 2 demonstrated better fit on every absolute and incremental index and was also more parsimonious.

**Table 3:** Descriptive statistics and normality indicators for teacher victimization, psychological well-being, and critical thinking variables.

Fit index	Criterion	Model 1 (full CT)	Model 2 (skills-only CT)
χ^2^ (df)	–	1982.55 (132) ***	1331.66 (101) ***
CFI	≥ 0.90	0.88	**0.91**
TLI	≥ 0.90	0.86	**0.90**
RMSEA (90% CI)	< 0.08	0.12 (0.11–0.12)	0.11 (0.10–0.11)
SRMR	< 0.08	0.0642	**0.0546**
AIC	lower = better	2096.55	**1433.66**

***p < 0.001. Bolded values indicate the model demonstrating superior fit relative to the comparison model based on conventional fit criteria.

Although neither model met the conventional RMSEA < 0.08 criterion, *Model 2’s* incremental indices (CFI/TLI ≥ 0.90) and lower SRMR indicate an acceptable representation given the complexity of the measurement model. Standardized factor loadings are presented in Table 4.

**Table 4:** Standardized factor loadings (λ) for model 1 and model 2.

Latent factor	Indicator	Model 1 λ	Model 2 λ
Teacher victimization	Parental TV	0.754	0.754
	Property TV	0.775	0.775
	Sexual TV	0.826	0.826
	Cyber TV	0.780	0.780
	Verbal TV	0.790	0.790
	Social TV	0.677	0.677
	Physical TV	0.739	0.739
Critical thinking	Critical openness	0.592	–
	Reflective skepticism	0.589	–
	Interpretation	0.844	0.839
	Analysis	0.919	0.918
	Evaluation	0.944	0.946
	Drawing conclusions	0.956	0.959
	Explanation	0.915	0.914
	Self-regulation	0.891	0.888
Psychological well-being	Negative emotions	0.677	0.677
	Positive emotions (rev.)	−0.953	−0.954
	Flourishing (rev.)	−0.523	−0.522

Results demonstrated that seven first-order victimization indicators loaded strongly on the second-order TV factor in both models (standardized loadings = 0.68–0.83). In *Model 1*, the six critical thinking skills indicators loaded very highly (*β* = 0.84–0.96) whereas the two dispositions were moderate (*β* ≈ 0.59). Removing the weak indicators in *Model 2* increased the average standardized loading for the remaining skills (*β* = 0.84–0.96 → 0.84–0.96; median = 0.93) and substantially reduced their residual variances. As expected, negative emotions loaded positively on the latent negative psychological well-being factor (*β* = 0.68), whereas positive emotions (*β* = −0.95) and flourishing (*β* = −0.52) loaded negatively, reflecting reverse-scored contributions, and factor loadings were stable across models. Structural path estimates are presented in Table 5.

**Table 5:** Structural path estimates for models 1 and 2.

Predictor → Outcome	B (SE)	CR	*p*	β	Model
Teacher victimization → Critical thinking	0.076 (0.038)	2.02	0.044	0.067	1
	0.195 (0.094)	2.08	0.038	0.068	2
Teacher victimization → Psychological ill-being	0.454 (0.062)	7.37	<0.001	0.261	1
	0.454 (0.062)	7.34	<0.001	0.261	2
Critical thinking → Psychological ill-being	−0.420 (0.055)	−7.67	<0.001	−0.277	1
	−0.163 (0.021)	−7.79	<0.001	−0.268	2

On the whole, eliminating the dispositional critical thinking indicators produced a model that fit the data better by every index examined (ΔCFI = +0.03; ΔAIC = −662.9), including reduced standardized residuals (SRMR = 0.0642 → 0.0546). The two dispositional indicators (critical openness, reflective skepticism) displayed the weakest factor loadings (*β* ≈ 0.59), large residual error variances, and numerous cross-indicator modification indices (e.g., MI = 288.43 for the residual covariance between Explanation and Conclusion), and their exclusion improved both fit and parsimony.

### Main effects and mediation analysis

Results showed that teacher victimization has a moderate direct adverse effect on psychological well-being (*β* = 0.26), so H1 was confirmed. Greater teacher victimization was associated with higher psychological ill-being, including diminished flourishing, reduced positive emotions and increased negative emotions. The effect size was in the small-to-moderate range but well within the bounds reported in previous studies.

Nevertheless, H2, which hypothesized that teacher victimization would be negatively associated with critical thinking, was not supported. On the contrary, a small positive association was observed between teacher victimization and critical thinking skills (*β* = 0.07). Furthermore, higher levels of critical thinking were associated with lower psychological ill-being (*β* ≈ −0.27), thereby supporting H3.

The results also confirmed H4: the indirect pathway from victimization to negative psychological well-being through CT was *negative* (*β* ≈ −0.02), indicating a very small indirect effect via critical thinking. The negative indirect effect shows a *partial mediation*: victimization slightly elevates critical-thinking skill, which in turn reduces negative psychological well-being.

Regarding the variance, in *Model 2*, the predictors accounted for 11% of the variance in critical thinking (R^2^ = 0.11) and 14% of the variance in psychological distress (R^2^ = 0.14). The corresponding values in *Model 1* were nearly identical (10 and 13%, respectively). To sum up, the data shows that enhancing critical-thinking skills may contribute modestly to lower psychological ill-being through an indirect pathway.

## Discussion

The present study sought to clarify the interplay between teacher victimization, psychological well-being, and critical thinking by testing a mediation model in which critical thinking operates as a mechanism linking victimization experiences to negative psychological well-being. Overall, the results provide partial support for the hypothesized model and offer several important insights into how cognitive resources are related to teachers’ responses to adverse experiences.

First, in line with H1, teachers who reported higher levels of victimization also reported greater psychological ill-being, characterized by lower flourishing and positive affect, and higher negative emotions. This finding is consistent with previous research showing that exposure to harassment, intimidation, or disrespect in school settings erodes teachers’ emotional resources and contributes to psychological strain (Olivier et al., 2021; McMahon et al., 2023). The observed association also aligns with evidence linking victimization to burnout (Ghadban et al., 2023), job dissatisfaction (Corbett et al., 2022; Moon et al., 2021), lower teaching efficacy (Yang et al., 2022), and intentions to leave the profession (McMahon et al., 2022; Moon and McCluskey, 2018; Olivier et al., 2021). Although the effect size in the current study is modest, it clearly identifies teacher victimization as a meaningful occupational stressor with implications for teachers’ psychological health and long-term retention.

Second, the study examined the associations between victimization and critical thinking (H2), and between critical thinking and psychological well-being (H3), as well as the indirect pathway proposed in H4. Contrary to H2, victimization was not negatively related to critical thinking; instead, a small but statistically significant positive association emerged. Teachers who reported more frequent victimization experiences also tended, on average, to report slightly higher critical thinking skills. This pattern is noteworthy because it provides the first empirical indication—within a school context—that there may be a systematic link between victimization and critical thinking, a relationship that previous work had not addressed (Pivorienė et al., 2024).

One possible explanation for this unexpected association can be drawn from post-traumatic growth and stress-related growth frameworks (Tedeschi and Calhoun, 1996; Calhoun and Tedeschi, 2006; Linley and Joseph, 2004). These perspectives suggest that, under certain conditions, exposure to challenging or adverse experiences can stimulate deeper reflection, cognitive restructuring, and the development of new perspectives. In the context of teaching, experiences of victimization may compel educators to re-evaluate their professional environment, reconsider interpersonal dynamics, and examine institutional norms and their own coping strategies. This reflective process may encourage more frequent use of analytical and evaluative reasoning—key components of critical thinking—as teachers attempt to make sense of and respond to complex social situations. The current findings can therefore be interpreted as tentative evidence that confrontation with adversity may, for some teachers, be associated with greater engagement in critical analysis and reflection.

At the same time, H3 was supported: higher levels of critical thinking were associated with lower psychological ill-being. Teachers who reported stronger critical thinking skills also reported less negative affect and higher flourishing and positive emotional experience (given the reverse scoring of those scales). This association is consistent with broader literature indicating that cognitive resources—such as the capacity to analyze situations, consider alternatives, and question assumptions—can promote more adaptive coping and reduce vulnerability to stress (Tiruneh et al., 2017). For teachers, critical thinking may help in reappraising difficult situations, generating constructive responses, and integrating challenging experiences into a coherent professional narrative, thereby supporting emotional stability and ongoing development (Wang and Jia, 2023). In this sense, the present findings extend prior work by demonstrating that critical thinking is not only a pedagogical competence but also closely linked to indicators of psychological well-being.

Third, the mediation analysis (H4) showed that critical thinking partially mediated the relationship between victimization and psychological ill-being, although the indirect effect was very small. Specifically, victimization was positively associated with critical thinking, and critical thinking, in turn, was negatively associated with ill-being, resulting in a slight negative indirect path from victimization to ill-being through critical thinking. In other words, within this sample, higher victimization was accompanied by a small increase in reported critical thinking skills, which was related to slightly lower psychological distress.

It is important to emphasize that this pattern reflects a mediated association. The study did not examine whether the strength of the victimization–well-being relationship varies at different levels of critical thinking; rather, it tested whether critical thinking lies on the pathway linking these constructs. The small negative indirect effect suggests that critical thinking may function as a cognitive process through which some teachers engage with and make sense of victimization experiences, and that this process is modestly related to better psychological outcomes. However, given the small magnitude of the indirect effect and the cross-sectional design, this result should be interpreted cautiously and viewed as a preliminary indication of a potential mechanism rather than evidence of a strong protective or conditional effect.

Taken together, the findings portray a nuanced pattern of associations among victimization, critical thinking, and psychological well-being. Victimization is clearly associated with elevated psychological distress, reinforcing its status as a harmful occupational stressor. Critical thinking, in turn, is robustly associated with better psychological outcomes, highlighting its potential importance for teachers’ mental health and professional functioning. The small positive association between victimization and critical thinking, combined with the negative link between critical thinking and ill-being, produces a weak but statistically significant mediated pathway, suggesting that cognitive processes may play a role in how victimization relates to psychological functioning.

From a practical standpoint, these results underscore the importance of reducing teacher victimization as a priority for educational policy and school management. At the same time, they suggest that professional development initiatives aimed at strengthening teachers’ critical thinking skills may contribute to better psychological outcomes, in part because such skills are associated with more favorable patterns of emotional functioning. Future research should investigate these relationships using longitudinal designs to clarify the temporal ordering of victimization, critical thinking, and well-being, and to determine whether changes in critical thinking over time help explain changes in psychological health. Qualitative studies could also illuminate how teachers themselves describe the role of reflective and analytical thinking in coping with experiences of victimization.

### Limitations and future directions

While this study provides valuable insights into the relationships among teacher victimization, psychological well-being, and critical thinking, several limitations should be acknowledged. First, the cross-sectional design limits causal inference; longitudinal or experimental studies are needed to better establish the directionality and temporal dynamics of these relationships. Second, the sample, although relatively large and diverse in terms of age and teaching experience, was predominantly female (90.1%). However, this distribution closely reflects the broader population of general education teachers and school leaders in Lithuania. Therefore, while gender imbalance was present, it mirrors the actual demographic composition of the profession and may not necessarily be considered a limitation. Even so, future research could aim to recruit more gender-diverse samples to explore potential gender differences in victimization experiences and outcomes.

Third, all data were obtained through self-report questionnaires, which are inherently vulnerable to biases such as social desirability and recall inaccuracies. This limitation is particularly salient in relation to the assessment of critical thinking, a complex and multidimensional construct. The reliance on a self-report measure may not adequately capture individuals’ actual critical thinking abilities, as such instruments primarily reflect subjective self-perceptions rather than objectively demonstrated cognitive performance. Accordingly, the associations observed among critical thinking, psychological well-being, and teacher victimization should be interpreted with appropriate caution. Future research would benefit from employing objective or performance-based measures of critical thinking to enhance the construct validity of assessments and to provide a more accurate representation of teachers’ cognitive competencies in real-world educational contexts.

Fourth, the study’s focus on teachers in Lithuania raises questions regarding the cultural specificity of the findings. Forms and prevalence of teacher victimization, as well as cultural conceptions of critical thinking, may differ across countries and educational systems. Consequently, cross-cultural replication studies are needed to test the external validity of these results and to assess whether similar patterns hold in other sociocultural contexts.

Additionally, the exclusion of the two dispositional indicators of critical thinking—critical openness and reflective skepticism—in Model 2 warrants theoretical consideration. Dispositional components represent an individual’s enduring tendency or motivation to engage in critical thought, whereas cognitive skill components reflect the operational execution of those abilities in practice. By omitting the dispositional indicators, Model 2 focuses more narrowly on the cognitive-skill dimension of critical thinking (e.g., interpretation, analysis, and evaluation), potentially providing a clearer assessment of the functional aspects of critical reasoning. However, this decision also narrows the construct’s conceptual scope, limiting the extent to which the model captures the attitudinal and motivational facets that are integral to comprehensive conceptualizations of critical thinking. Consequently, the findings derived from Model 2 should be interpreted as reflecting the cognitive rather than dispositional aspects of critical thinking. Future research should aim to integrate both dispositional and cognitive dimensions within more refined measurement models to capture the full complexity of teachers’ critical thinking and its role in psychological well-being and victimization processes.

## Conclusion

This study highlights the significant adverse impact of teacher victimization - encompassing verbal, social, cyber, physical, and other forms of aggression - on educators’ psychological well-being, confirming that higher victimization is linked to increased psychological distress. Contrary to initial expectations, teacher victimization was not associated with diminished critical thinking; instead, a slight positive relationship emerged, suggesting that exposure to victimization may, in some cases, stimulate certain critical thinking skills.

Importantly, critical thinking was found to function as a cognitive mechanism linking victimization experiences to psychological ill-being, as stronger critical thinking abilities predicted lower psychological ill-being. The partial mediation effect indicates that critical thinking explains a small portion of the association between victimization and teachers’ psychological ill-being through an indirect pathway, contributing modestly to lower distress.

These findings underscore the potential value of fostering critical thinking skills among educators as part of interventions aimed at reducing the psychological toll of workplace victimization. Future research should further investigate how critical thinking develops in response to stressors and explore targeted training programs that enhance these skills to promote teacher resilience and well-being.

## Data Availability

The raw data supporting the conclusions of this article will be made available by the authors, without undue reservation.
